# Inferring Fish Escape Behaviour in Trawls Based on Catch Comparison Data: Model Development and Evaluation Based on Data from Skagerrak, Denmark

**DOI:** 10.1371/journal.pone.0088819

**Published:** 2014-02-20

**Authors:** Ludvig Ahm Krag, Bent Herrmann, Junita Diana Karlsen

**Affiliations:** 1 DTU Aqua, National Institute of Aquatic Resources, North Sea Science Park, Hirtshals, Denmark; 2 SINTEF, Fisheries and Aquaculture, Fishing Gear Technology, North Sea Science Park, Hirtshals, Denmark; Aristotle University of Thessaloniki, Greece

## Abstract

During the fishing process, fish react to a trawl with a series of behaviours that often are species and size specific. Thus, a thorough understanding of fish behaviour in relation to fishing gear and a scientific understanding of the ability of different gear designs to utilize or stimulate various behavioural patterns during the catching process are essential for developing more efficient, selective, and environmentally friendly trawls. Although many behavioural studies using optical and acoustic observation systems have been conducted, harsh observation conditions on the fishing grounds often hamper the ability to directly observe fish behaviour in relation to fishing gear. As an alternative to optical and acoustic methods, we developed and applied a new mathematical model to catch data to extract detailed and quantitative information about species- and size-dependent escape behaviour in towed fishing gear such as trawls. We used catch comparison data collected with a twin trawl setup; the only difference between the two trawls was that a 12 m long upper section was replaced with 800 mm diamond meshes in one of them. We investigated the length-based escape behaviour of cod (*Gadus morhua*), haddock (*Melanogrammus aeglefinus*), saithe (*Pollachius virens*), witch flounder (*Glyptocephalus cynoglossus*), and lemon sole (*Microstomus kitt*) and quantified the extent to which behavioural responses set limits for the large mesh panel’s selective efficiency. Around 85% of saithe, 80% of haddock, 44% of witch flounder, 55% of lemon sole, and 55% of cod (below 68 cm) contacted the large mesh panel and escaped. We also demonstrated the need to account for potential selectivity in the trawl body, as it can bias the assessment of length-based escape behaviour. Our indirect assessment of fish behaviour was in agreement with the direct observations made for the same species in a similar section of the trawl body reported in the literature.

## Introduction

During the last decade, advanced and sophisticated trawl designs have been developed in an attempt to reduce by-catch in the commercial fishing industry. The major challenge facing trawl designers is to improve selectivity, typically for one or two focus species, while maintaining high catch efficiency for the target species and sizes. The process by which fish are caught in a trawl involves a sequence of behavioural responses to the different stages of the catching process [1]. It is important to identify these behavioural patterns for relevant species and sizes and to define the factors that affect these patterns, as such knowledge would allow more directed development of economically profitable trawl systems with improved selectivity.

Extensive research has been focused on understanding fish behaviour in relation to fishing gears to aid the development of more efficient species or size selective fishing gears [1]–[3]. Behavioural patterns of several species have been described qualitatively for trawls at different stages of the catching process. The main conclusions are outlined and reviewed in [1] and [3]. There is an overall understanding of the behavioural pattern through the catching process in trawl gear for a few important commercial species such as haddock (*Melanogrammus aeglefinus*), cod (*Gadus morhua*), whiting (*Merlangius merlangus*), and some flatfish species [1], [3]–[4].

The understanding of fish behaviour is often synthesised from observations of different trawl designs in different fishing areas. However, a fish may behave differently when it encounters different trawl designs. Thus, there is a need to assess fish behaviour not in trawl gear in general but more specifically for a given design category. Ideally, fish behaviour, including intra-individual variation, should be mapped in a quantitative way for a given gear design in a given area under the conditions in which the gear is used. Observation cruises are expensive and observation conditions often are harsh. Poor, inconclusive, or biased results are often obtained, although the quality of underwater cameras and other observation equipment has improved greatly during the last decade [2], [5]. Another challenge is that optical observations can only be made during the day when there is sufficient light at observation depth. Commercial fishing is often conducted around the clock, and experimental fishing has demonstrated that fish behaviour in relation to fishing gear varies between day and night for several species [5]–[9].

Today, numerous types of optical and acoustic observation equipment and techniques are available to researchers. Acoustic techniques, which are independent of visibility and light at depth, still depend on optical methods for species recognition and therefore face the same limitations as optical observation techniques. In addition to optical or acoustic observations, fish behaviour can be inferred from the catch composition (e.g., by using spatially divided gear designs). Examples of such designs are separator trawls [9]–[11] and similar experimental designs in which the trawl body is divided into vertically separated collecting bags [5], [12]–[15]. However, installing separating panels or other separating devices inside the trawl body introduces new structures that can affect fish behaviour [5].

In this study, we evaluated the effect of inserting a large mesh panel on catch efficiency of five commercial species in the *Nephrops* (*Nephrops norvegicus*) directed fishery in Skagerrak off northern Denmark. This fishery is conducted in relatively deep waters on muddy grounds where optical observation techniques repeatedly have failed during our prior experiments in this area. This study was conducted without any direct observations of fish behaviour and without use of spatially divided gear designs. The study was based solely on analysis of catch data collected using a twin trawl in which the experimental trawl was equipped with an 800 mm diamond mesh panel in the top side of the entire aft tapered section of the trawl. We developed a new model to describe and quantify fish behaviour indirectly based on analysis of catch data alone. Using this method, we quantified the length-dependent behavioural response for cod, haddock, saithe (*Pollachius virens*), lemon sole (*Microstomus kitt*), and witch flounder (*Glyptocephalus cynoglossus*) in relation to the large mesh panel in the experimental trawl body.

## Materials and Methods

### Ethics statement

This study did not involve endangered or protected species. Experimental fishing was conducted onboard a Danish commercial trawler in accordance with the fishing permit granted by the Danish AgriFish Agency (J. no. 2004-243-120). No other permit was required to conduct the study.

### Experimental setup for data collection

**Figure 1:**
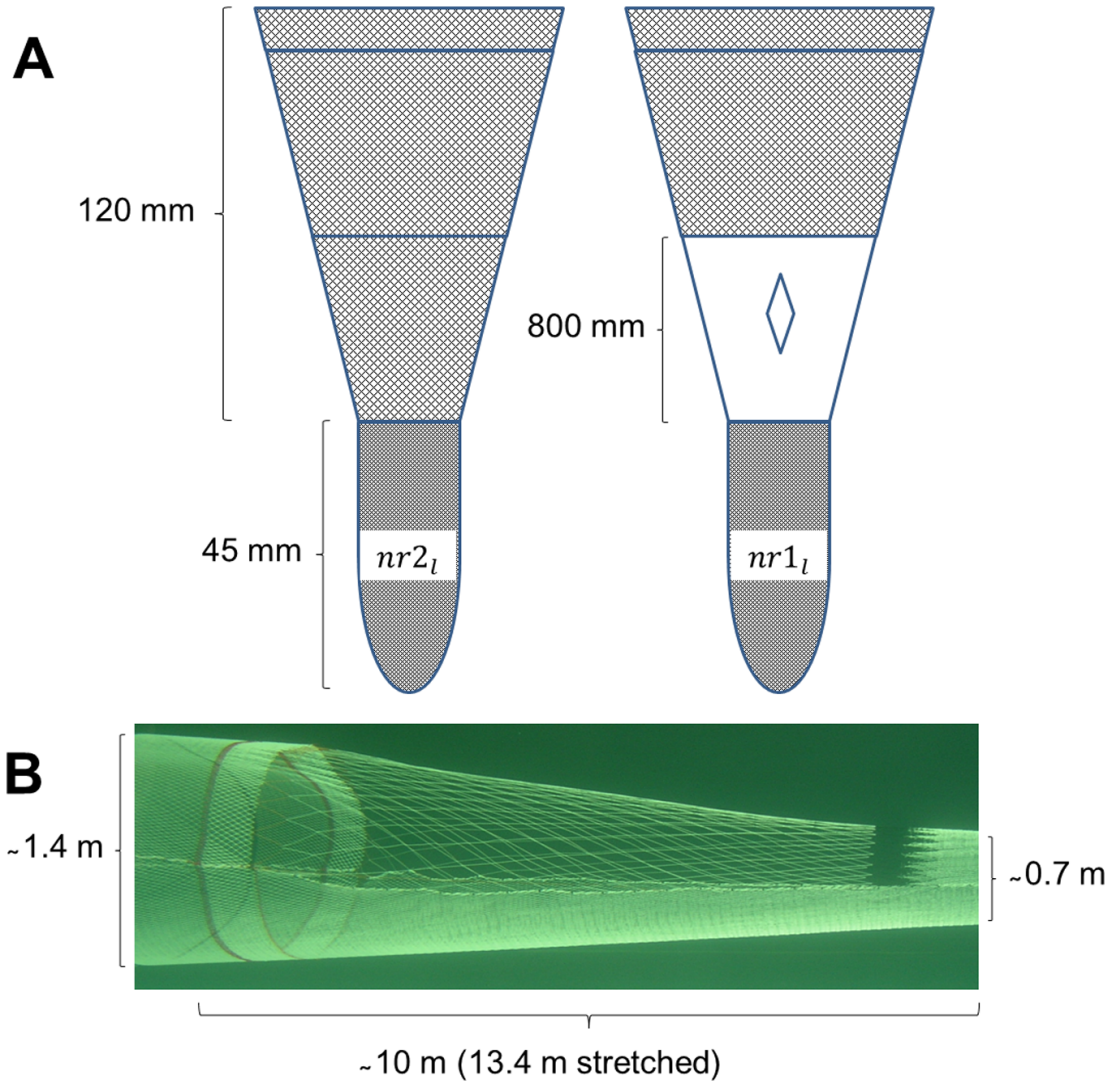
Experimental setup. (A) The upper panels in the experimental setup of the standard trawl (left) and the experimental trawl (right). The lower panels in both trawls are similar to the upper panel of the standard trawl. (B) The 800 mm large mesh panel inserted in an identical trawl design in a scale model (1∶8) in the flume tank.

Two identical *Cosmos Combi* trawls (540 meshes of 115 mm (PE) in the fishing circle circumference) were constructed. In the experimental trawl, an 800 mm diamond mesh panel was installed from selvedge to selvedge in the entire upper panel in the aft tapered section (13.8 m stretch length). The 800 mm panel was made of 6 mm single twine (PE). The joining ratio between the 115 mm and 800 mm meshes was 7∶1, except that every third 800 mm mesh was joined at a 6∶1 ratio. This joining ratio (7∶7:6) was used in order to obtain the same mesh opening angle in both the 115 mm and the 800 mm meshes. The extension and codend were made of 45 mm meshes in both trawls (Figure 1). Actual mesh sizes were measured prior to the experiment. The 800 mm mesh size in the large mesh panel could not be measured with the available mesh measurement tools and is therefore given as the nominal mesh size.

Experimental fishing was conducted aboard a commercial trawler (511 KW). The vessel’s twin trawl system with three towing warps was used. The twin rig was spread with two 3.73 m^2^ Thyborøn V-doors (type 11, standard) and a 1200 kg rolling centre clump. The sweeps were 204 m long single sweeps with a 5 m backstrop behind the doors. The trawl doors and clump were equipped with distance sensors, which provided information about the basic geometry of the front part of both trawls during towing. The total catch of fish was length measured to the nearest cm and *Nephrops* was measured to the nearest mm. For subsequent data analysis, 0.5 cm was added to each measured fish length and 0.5 mm to each measured *Nephrops* carapace length. All hauls were made during daylight hours between sunrise and sunset. The two trawls were interchanged halfway through the experiment to compensate for any systematic effects between the two gears.

### Catch comparison analysis

Figure 1 shows the experimental setup. The number of individuals in each length class collected in the two codends was used to evaluate the length-dependent relative catching efficiency of the two trawls by species. On a haul-by-haul basis, the experimental catch comparison rate, *rate_l_*, for each species was given by:
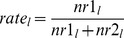
(1)where *nr1_l_* is the number of fish of length *l* of the given species collected in codend 1 and *nr2_l_* is the number collected in codend 2. In catch comparison analysis, the experimental *rate_l_* is often modelled by the function *rate(l)* of the following form [16]:
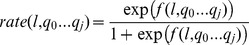
(2)where f is a polynomial of order j with coefficients *q_0_* to *q_j_*. Thus, rate(*l,q_0_…q_j_*) expresses the likehood of finding a fish of length l in the large mesh panel trawl codend given that it is found in one of the two codends. A value of 0.5 for rate would mean that the likelihood of finding the fish in one of the two codends is equally high, implying that introducing the large mesh panel in the trawl did not have any effect on the catch efficiency. On a haul-by-haul level, the values of the parameters describing *rate(l)* in formula (2) can be estimated by minimising the following equation, assuming that the model *rate(l)* adequately describes the catch comparison rate between the two trawls:
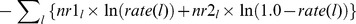
(3)where the summation is over the length classes in the experimental data.

To model the catch comparison *rate(l)* between the two trawls, we applied formula (2). We considered *f* up to an order of 4 with parameters *q_0_*, *q_1_*, *q_2_*, *q_3_*, and *q_4_*. Leaving out one or more of the parameters *q_1_…q_4_* led to an additional 31 models that were considered as potential models for the catch comparison *rate(l)* between the two trawls. Selection of the best model for *rate(l)* among the 32 competing models was based on a comparison of the AIC values for the models. The model with the lowest AIC value was selected [17].

Often the catch comparison curve is estimated for each haul separately, and then the results from single hauls are applied in a two-step procedure to estimate a mean curve while considering between-haul variations in the catch comparison rate [18]. However, in this study we did not have any particular interest in the between-haul variation in the catch comparison rate between the two trawls; instead we wanted to estimate an average catch comparison rate for the trawls based on all of the available hauls. Therefore, we used another approach that involved applying formula (3) summed over hauls and estimating an average curve based on formula (2). We used a double bootstrap approach with 2000 bootstrap repetitions to estimate the Efron percentile 95% confidence limits [19] for *q_0_…q_4_* and *rate(l)* for all relevant length values. This approach, which avoided underestimating confidence limits when averaging over hauls, is identical to the one described by Sistiaga et al. [20] and Herrmann et al. [21]. Traditionally, the confidence limits for a curve and for the parameter values describing this curve are estimated without accounting for potentially increased uncertainty resulting from uncertainty in selection of the model used to describe the curve [22]. We accounted for the additional uncertainty in the catch comparison curve by incorporating an automatic model choice that was based on which of the 32 models produced the lowest AIC into each of the 2000 bootstrap repetitions. The catch comparison analyses were performed using the software SELNET [20], [21], [23]–[25].

We were able to use the above described double bootstrap method for all species but lemon sole. For lemon sole, a single bootstrap technique that did not account for between-haul variation was used due to weak data at the haul level. Because all hauls were pooled, no hauls were excluded for lemon sole.

### Assessment of contact behaviour

A main aim of this work was to investigate the extent to which fish behaviour, in terms of their length-dependent contact with the large mesh panel, sets limits for the selective efficiency of the large mesh panel. Thus, we needed a model that, based on the catch comparison rate *rate_l_,* would enable us to estimate the likelihood that a fish that enters the large mesh section would contact the panel to escape. Due to the large mesh size in the panel, we assumed that every fish that actually contacted the panel escaped. Based on this assumption and restricting this part of the assessment to sizes of fish for which the large mesh panel is the only panel in the body of both the test and control trawl that potentially could release fish, the following relation was derived between the catch comparison rate and the length-dependent contact likelihood *c(l)* with the large mesh panel (see Appendix S1):
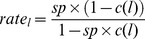
(4)where *rate_l_* can be obtained from (1). *sp* is the assumed length-independent entry likelihood (split) of a fish into the trawl containing the large mesh panel given that it enters one of the two trawls that were fished simultaneously. Thus, the likelihood of entering the standard trawl is *1.0 – sp*. *c(l)* is the length-dependent contact likelihood of a fish with the large mesh panel given that it enters the section in the experimental trawl where the large mesh panel was inserted. A flexible formula for *c(l)*, which enables modelling increasing, decreasing, and constant contact likelihood with the large mesh panel, is given by:
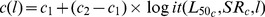
(5)where *c_1_* and *c_2_* are constants that both are constrained to the interval *[0.0;1.0]* and L50c is the midpoint fish length at which the value of the contact likelihood will be the mean of *c_1_* and *c_2_*. The value of *SRc* defines how quickly the contact shifts from a value close to *c_1_* to a value close to *c_2_* with increasing fish length in the vicinity of *L50c*. Thus, if the value of *SRc* is close to 0.0, the change in the contact likelihood will appear over a small length range, whereas a value far from 0.0 will result in a change that will cover a wider length span. Herein, we applied formula (5) to model the large mesh panel contact likelihood. Estimation of the parameter values of *c_1_*, *c_2_*, *L50c*, and *SRc* was conducted species by species by applying formula (5) for *c(l)* in formula (4) and then using *rate(l)* in (3), but the length classes used were constrained to the interval above which the 115 mm netting can be selective and below which the large mesh panel can begin to restrict escapement. In addition to this model (named *M1*) based on formula (5), we also considered three simpler models for the length-dependent large mesh panel contact (Table 1).

**Table 1:** Simpler models derived from model 1 (*M1*). See text for details.

Model name	Equation
*M2*	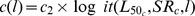
*M3*	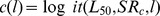
*M4*	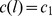

Selection of the best model among *M1, M2, M3*, and *M4* was carried out for each species individually by selecting the model that produced the lowest AIC value. Confidence intervals for the catch comparison curve *rate_l_* and for the large mesh contact curve *c(l)* were generated using the same double bootstrap technique described in the previous section.

Except for the large mesh panel, no other sections in the two trawl bodies had a mesh size that exceeded 115 mm. Therefore, for the assessment of the length-dependent contact likelihood with the large mesh panel, we needed to identify the potential size selection of the different species for netting with mesh size 115 mm to determine where to cut off the experimental data. To do this, we used realistic mesh openness based on flume tank measurements of the mesh openings in the net section of interest. We then applied the FISHSELECT methodology [26] to estimate the maximum size of each species that can penetrate such meshes (Table 2). The maximum mesh opening angle was found in the forward end of the panel with an opening angle of about 30°. The mesh opening angle was based on flume tank measurements of a 1∶8 scale model. Using the FISHSELECT software, we then estimated the maximum size of cod that can pass through a 115 mm mesh with an opening angle of 30°. For haddock we used values reported by Krag et al. [27], and for *Nephrops* we used values from Frandsen et al. [28]. We used unpublished morphology data for lemon sole. No morphology measurements were available for saithe and witch flounder, so we assumed that the morphology of saithe was similar to that of cod and that the morphology of witch flounders was similar to that of lemon sole. Single hauls with fewer than 10 individuals of each species were excluded from the analysis.

**Table 2:** Maximum lengths of fish that can escape through the 120

Species	Maximum escape length	Reference
Cod	33 cm	[26]
Haddock	33 cm	[27]
Saithe	33 cm	no data, used data for cod
*Nephrops*	all sizes can escape	[28]
Witch flounder	28 cm	no data, used data for lemon sole
Lemon sole	28 cm	unpublished data

The values were based on mesh opening measurements made from flume tank observations combined with morphology based estimates of selectivity.

### Simulation of the catch comparison curve

We suspected size selection was occurring in the standard trawl. To get an idea of what kind of curve we would expect for the catch comparison rate if there was size selection in the standard trawl, we conducted a simple parametric simulation to estimate the theoretical catch comparison *rate_l_*. We used the parametric simulation function built into the software SELNET to model equations (A8) and (A11) in Appendix S1. In the simulation, we assumed that the likelihood of fish contacting the upper panel in the large mesh section had the same length dependency for both trawls. We assumed that the fish try to stay clear of the netting in the upper panel by maintaining distance from it. Such behaviour will result in a low level of contact with the large mesh panel. The large mesh panel is situated in the last tapered section, which dramatically narrows in the volume of the trawl body. We therefore expected that most fish came in contact with the large mesh panel and escaped unless they actively swam away from the panel. We also assumed that this behaviour depended on the size of the fish, as size is related to swimming ability. As an example, we simulated that 50% of the small fish (below 40 cm) would come in contact with the large mesh panel. For larger fish (40 to 80 cm) we assumed a reduction in their contact likelihood, and for the largest fish (above 80 cm) we assumed that the contact likelihood would be nearly zero. This kind of behavioural modelling can be done using formula (5) by selecting specific values for the model parameters. We used this in the SELNET simulation with the following parameter values: *c_1_ = 0.5*, *c_2_ = 0.0*, *L50_c_ = 65 cm*, and *SR_c_ = 15 cm* (parameters in formula 5). For the fish that contacted the panel netting and thus had a length-dependent chance of escaping through it, we assumed that the process could be modelled by a *logit* function with parameters *L50_p_* and *SR_p_* [29]. For the 115 mm panel we assumed *L50_p_ = 30 cm* and *SR_p_ = 5 cm*, whereas for the large mesh panel we used values that would result in the release of cod of every size that were simulated to contact the panel (*L50_p_>>115 cm*). We assumed that the entry of fish into the two trawls was equally likely (*sp* = 0.5). To make the simulations as realistic as possible, we applied a size structure similar to the one observed in the experimental data for cod. The SELNET simulation resulted in a virtual population for cod, which then was analysed in SELNET using the same method that was applied for the experimental data (see catch comparison analysis).

## Results

### Catch comparison analysis

**Figure 2:**
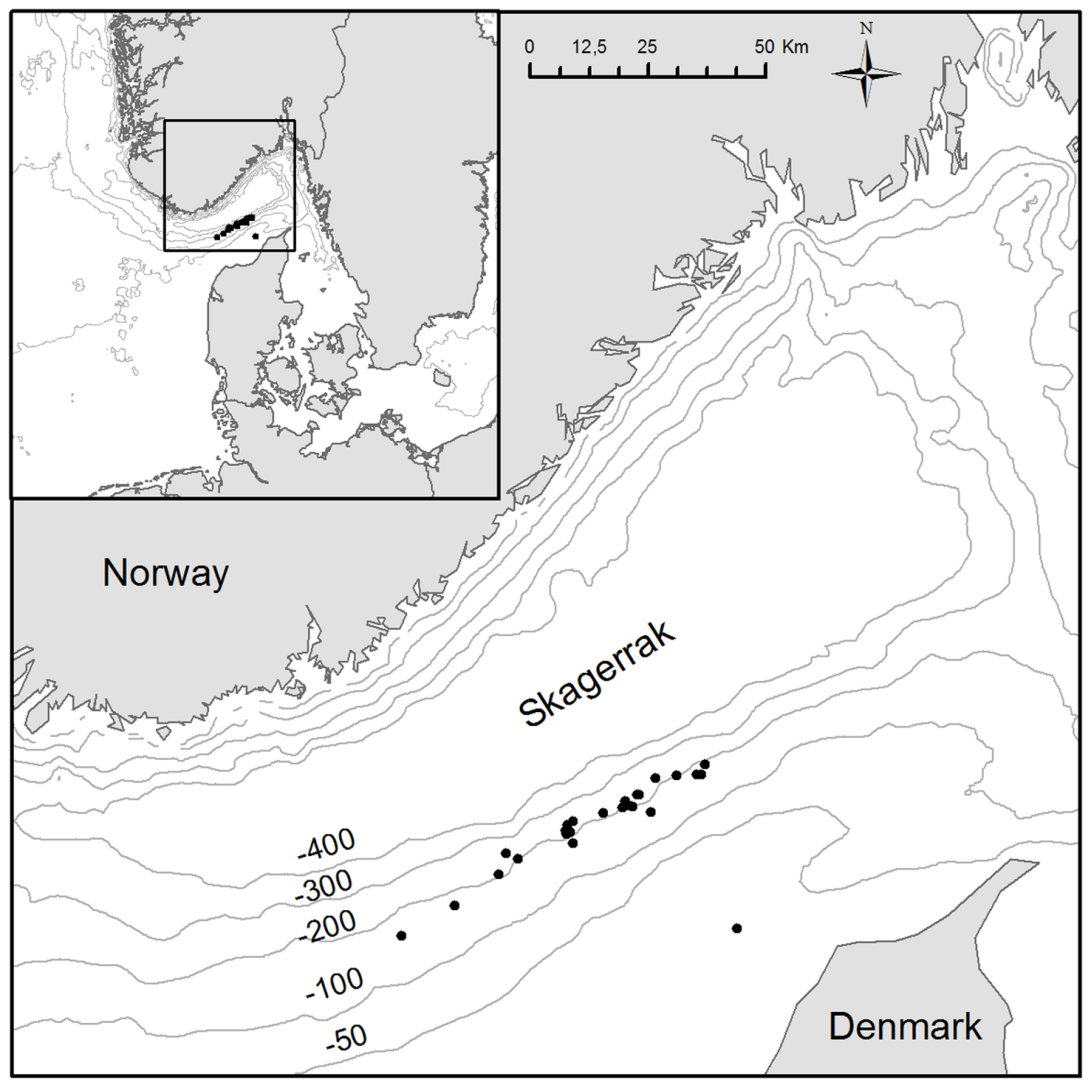
A map of Skagerrak showing the starting position of each trawl tow (black dots).

Table 3 lists the measurements of mesh size in the codend and large mesh panel. All measurements were conducted on dry netting prior to the cruise. A total of 25 valid hauls were conducted in June in Skagerrak on commercial grounds typically used by the Danish mixed species fleet (Figure 2). Towing time was 3 h. Additional operational conditions are summarised in Table 4. Cod, haddock, saithe, *Nephrops*, lemon sole, and witch flounder were caught in reasonable numbers and included in the analysis. All *Nephrops* were measured, except for in haul no. 8, which was subsampled due to large catch size. In this haul, 41% of the individuals were measured in the control trawl and 47% in the experimental trawl.

**Table 3:** Nominal and measured mesh sizes for the standard and experimental trawls.

Trawl	Gear section	Nominal meshsize (mm)	No. of meshesmeasured	Average mesh size (mm) ± SD	
				ICES 4 kg	EU 5 kg
Experimental	Large mesh panel	800	*	*	*
Experimental and standard		120	50	115.35±2.56	119.79
Experimental	Codend	42	50	41.39±1.10	43.05
Standard	Codend	42	50	41.66±0.85	43.33

*The instruments available for measuring meshes in trawls were not capable of measuring such large meshes.

**Table 4:** Operational conditions during experimental fishing.

	Depth (m)	Door spread (m)	Wire length (m)	Speed (knots)	Wind (m/s)
Average	169.49±35.69	200.78±14.92	514.80±64.09	2.85±0.20	4.44±4.27
Min–Max	24.6–213.8	144.6–210.4	232–556	2.0–3.2	0–16

**Figure 3:**
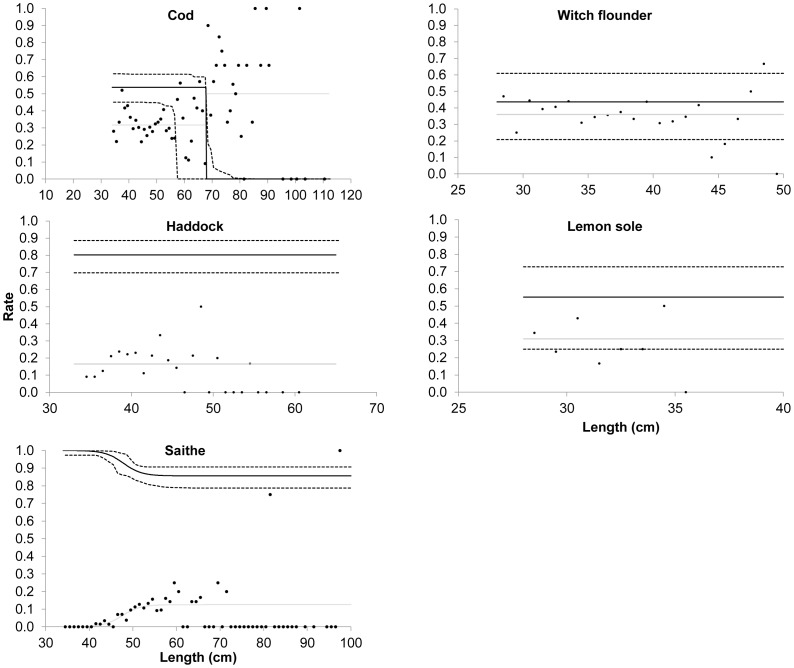
Catch comparison analysis and populations retained in both the experimental and standard trawls. Solid lines are mean estimates, and dotted lines indicate 95% confidence limits.

The large mesh panel significantly increased the escapement, and thus reduced the catch, of saithe, haddock, cod, witch flounder, and lemon sole, as indicated by the catch comparison rate being significantly lower than 0.5 for a large range of length classes (Figure 3). Among the gadoids, the effect was largest for saithe and smallest for cod. There was no significant difference in catches between the standard and experimental trawls for *Nephrops* above 38 mm carapace length, as 0.5 was within the confidence limits for these length classes. The experimental trawl caught significantly fewer *Nephrops* with carapace length ranging from 25 to 38 mm (Figure 3). The fit statistics showed that the model applied described the experimental data sufficiently well, as the model’s P-values were >0.05 for all species except for cod (Table 5). In the residuals [29] for cod, no structure was detected in the deviations between data and the model. Therefore, we were confident in applying the model for all species investigated.

**Table 5:** Fit statistics for the catch comparison analysis. DOF = degrees of freedom.

Species	P-value	Deviance	DOF
Saithe	0.9511	47.35	65
Haddock	0.6622	37.65	42
Cod	0.0053	114.74	79
Witch flounder	0.9733	15.45	28
Lemon sole	0.2661	25.67	22
*Nephrops*	0.6765	41.12	46

The catch comparison curve for cod and haddock was cup shaped, which, when interpreted as contact, would mean that the medium sized fish in Figure 3 were more likely to escape than smaller and larger individuals. This result contradicts our expectation of a constant or monotonic progression of the catch comparison curve, which would indicate that the escape behaviour of fish gradually changed over length, or, alternatively, no length-dependent effect.

### Assessment of contact behaviour

Table 2 lists the length classes that could pass through the 115 mm mesh size and thus were excluded from analysis. For cod and haddock, this led to the exclusion of a large proportion of the caught populations from the analysis due to large numbers of relatively small individuals in the catch. In contrast, only a few individuals were excluded for saithe, lemon sole, and witch flounder. All sizes of *Nephrops* could escape through the 115 mm meshes, thus *Nephrops* was not included in this part of the analysis.

**Figure 4:**
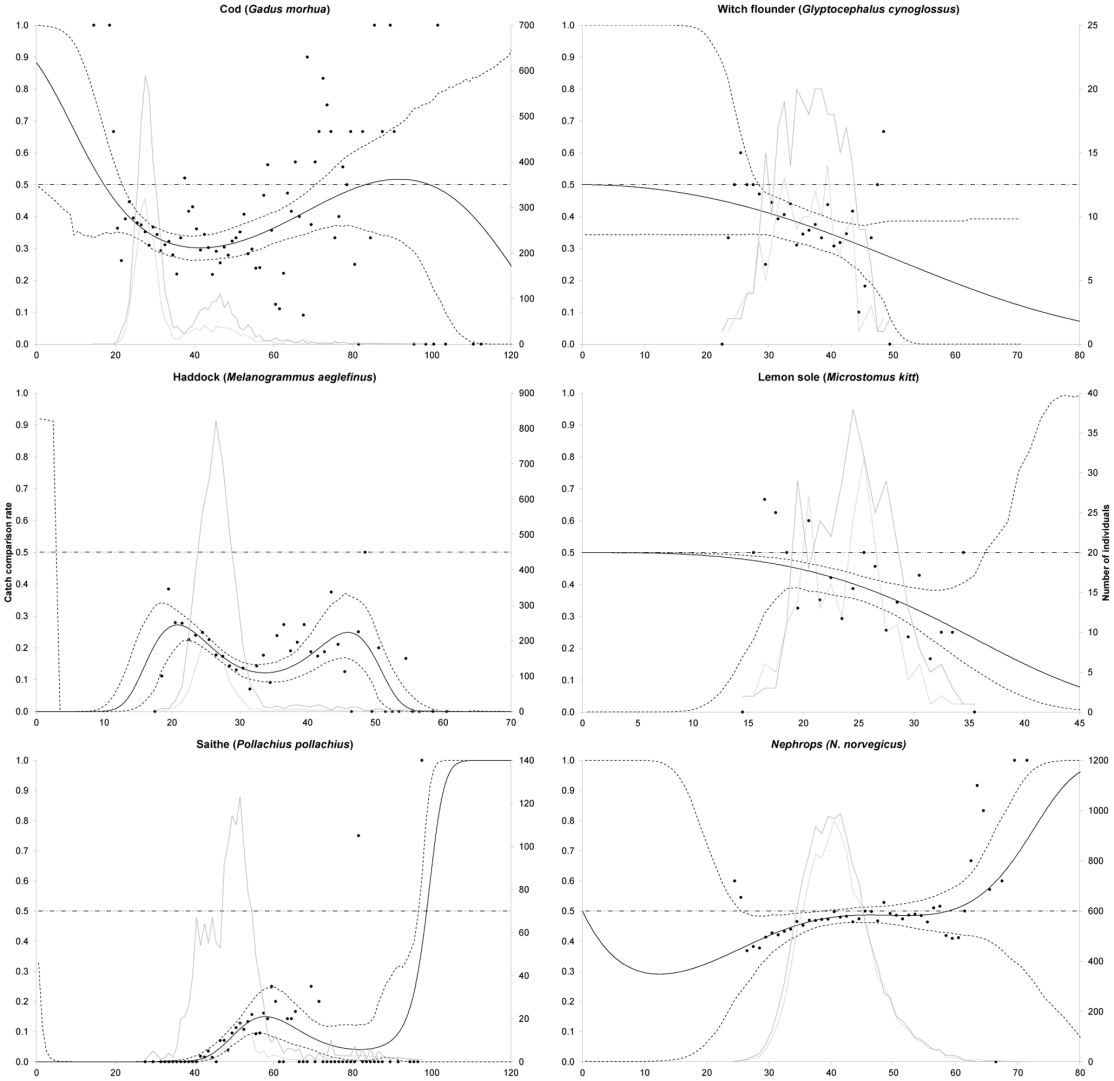
Estimated average escape behaviour (contact rate) (solid black curve) ±95% confidence limits (broken black curves), estimated mean retention (grey curve), and length-based retention data (black dots). Only length classes included in the catch and that could not escape through the 120

The model (*M1* to *M4*) with the lowest AIC value was chosen to describe the experimental data (Table 6). All saithe below 40 cm and about 85% of the larger sized fish escaped through the large mesh panel (Figure 4). About 80% of haddock, 44% of witch flounder, and 55% of lemon sole escaped through the large mesh panel, and no length dependency was observed. The length-dependent escape curves for saithe, haddock, witch flounder, and lemon sole all exhibited a constant or gradual monotonic progression (Figure 4).

**Table 6:** Fit statistics and choice of model.

		Cod	Haddock	Saithe	Witch flounder	Lemon sole
Hauls excluded		none	1,2,3,4,5,7,8,9,19,21,24	8,21	5,6	none
Length range (cm)		34–112	34–61	34–112	28–50	28–35
AIC	M1	2618.49	205.33	**873.26**	600.30	111.73
	M2	**2616.49**	205.57	918.15	599.65	109.73
	M3	2628.93	203.57	916.15	597.65	107.73
	M4	2631.82	**203.14**	931.76	**596.84**	**105.94**
P-value	M1	0.0098	0.8319	0.6535	0.9018	0.6698
	M2	0.0124	0.7623	0.0006	0.8794	0.7839
	M3	0.0008	0.8073	0.0008	0.9103	0.8663
	M4	0.0004	0.7731	0	0.8963	0.9057
Deviance	M1	84.82	15.67	52.25	11.60	3.20
	M2	84.82	17.91	99.14	12.95	3.20
	M3	99.27	17.91	99.14	12.95	3.20
	M4	104.15	19.47	116.75	14.14	3.41
DOF	M1	57	22	57	19	5
	M2	58	23	58	20	6
	M3	59	24	59	21	7
	M4	60	25	60	22	8

Hauls that were excluded from the analysis due to low number of individuals are listed for the individual species.

The model used in subsequent analysis is indicated in bold. For further description of models (M1–M4), see the text.

The pattern for cod differed from those of the other fish species. Figure 4 illustrates a knife-edge change in the mean length-dependent escape curve for cod at one specific length (68 cm). This is unexpected from a biological point of view and gives an unrealistic description of cod escape behaviour.

### Simulation of the catch comparison curve

**Figure 5:**
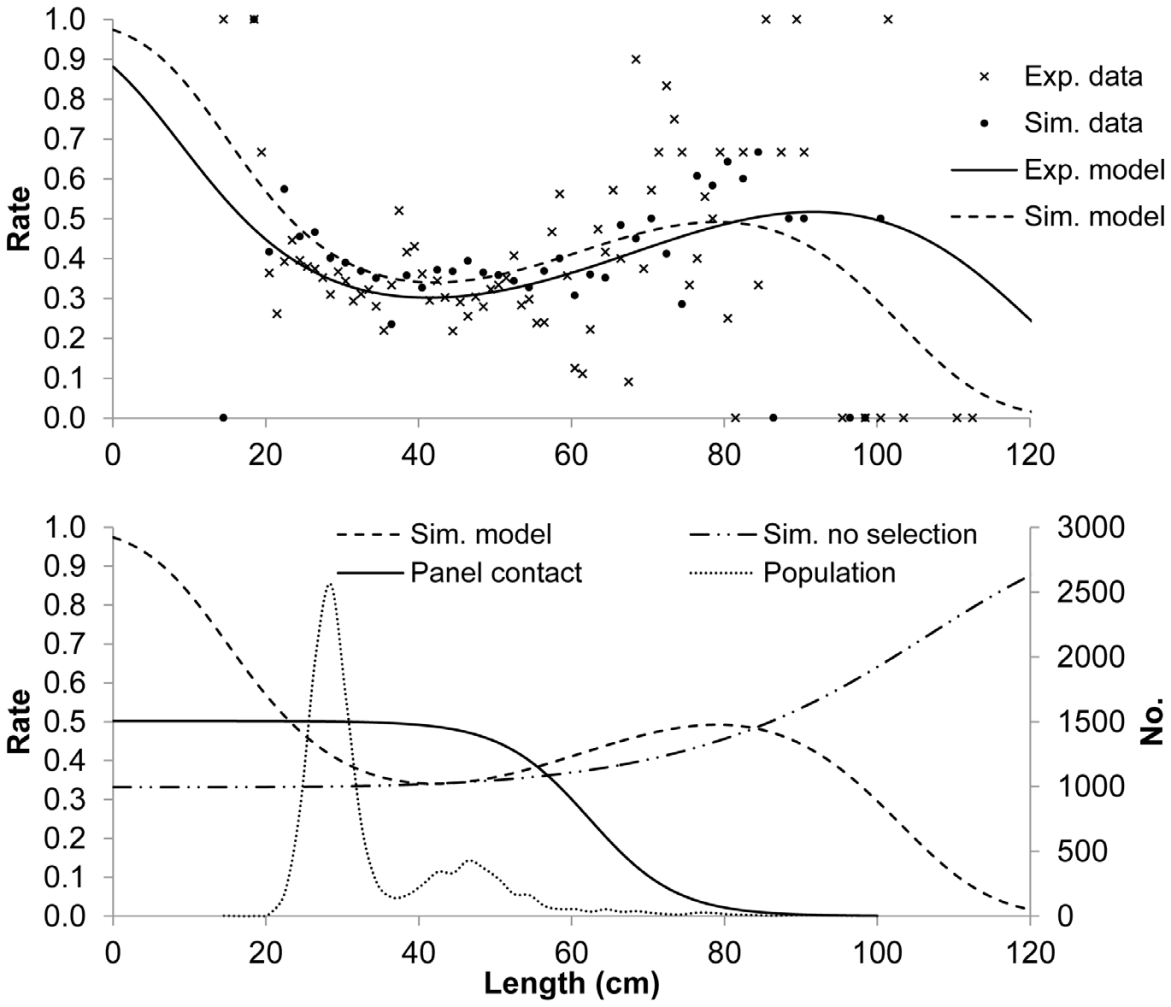
Catch-comparisons. Top: Experimental data showing unexpected cup-shaped structure for cod escape behaviour and simulated data using the same population assuming selectivity in the 120 mm mesh panel of the standard trawl that corresponds to the large mesh panel in the experimental trawl. Bottom: Models assuming presence (same curve as in the top plot) and absence of selectivity in the 120 mm mesh panel of the standard trawl that corresponds to the large mesh panel in the experimental trawl based on simulated data similar to the top plot. The contact with the large mesh panel (panel contact) is also included.

The curves for the experimentally obtained catch comparison rate for cod (shown in Figure 3) and the theoretical catch comparison rate (*rate_l_*) from the simulation assuming size selection in the standard trawl both were cup shaped (Figure 5, top). Thus, size selection in the standard trawl could explain the cup-shaped nature of the catch comparison curve and was considered to be a more plausible explanation than similar escape behaviour for the observed difference between medium sized cod vs. small and large cod. To further demonstrate that size selection in the standard trawl resulted in the cup-shaped catch comparison curve, we conducted an additional simulation in SELNET with the same parameters, except that we used values of *L50_p_* and *SR_p_* that simulated no selection in the panel in the standard trawl. Results from this simulation showed that the cup-shaped nature of the curve disappeared (Figure 5, bottom). Almost no fish outside the range of 20 to 80 cm were present in the simulation (population structure given in Figure 5), and therefore the actual shape of the catch comparison curves should not be applied outside this range. Figure 5 (bottom) also shows decreasing panel contact with increasing fish size. Thus, the simulation analysis revealed that two factors affected the nature of the catch comparison curve in this study: fish behaviour in relation to the large mesh panel and the selective properties in the corresponding gear section in the standard trawl.

## Discussion

In this study, we quantified the length-dependent escape behaviour of five commercial fish species in the mixed demersal fishery in Skagerrak in terms of their contact likelihood in relation to a large mesh panel placed in the aft part of a trawl. This assessment of fish behaviour was made without using optical or acoustic observation methods and without dividing the trawl gear into different compartments, which potentially could affect the behaviour of the fish species in question. The indirect method applied in our approach is based on catch data and can therefore potentially include every fish in the analysis, in contrast to optical observation techniques [5], and can be used under all physical conditions (e.g., independent of light and turbidity levels). Furthermore, our approach enabled us to describe escape behaviour over a large section in the main body of a trawl for all species caught and included uncertainties of the estimates, which is not possible with direct observation techniques. The method developed in this study can be used to survey escape behaviour along the full length of a trawl. This type of survey could provide detailed quantitative descriptions of behaviour, including uncertainties about the estimates, from the main body of the trawl, which can be difficult to collect with direct observation techniques. A further advantage of the described method is that detailed information about fish behaviour can be collected at low cost and during codend selectivity studies. Sections of large meshes in the forward part of the trawl are commonly used, especially in pelagic and semi-pelagic fisheries, to guide fish into the narrower and smaller meshed aft part of the trawl. However, little quantitative information is available about the guiding effect of large meshes in the forward part of trawls. The method presented herein could be used to conduct quantitative studies of escape behaviour in these very large trawls.

The catch comparison analysis revealed a cup-shaped catch comparison curve for cod and haddock. If these curves are interpreted solely as escape behaviour, the results suggest increasing escape behaviour for the smaller individuals (cod>40 cm, haddock >34 cm) and decreasing escape behaviour for the large individuals (cod <40 cm, haddock <34 cm). To avoid misinterpretation in such analysis, it is important to understand, and subsequently account for, additional selectivity that occurs in the section of the standard trawl (115 mm) that corresponds to the large mesh panel in the experimental trawl. Misinterpretation can be avoided by reducing the mesh size to a small non-selective mesh size. One consequence of using a large commercial mesh size (115 mm) was that we had to exclude all individuals in the population that were able to escape through it. This weakened the data, as observed for cod in the current study. Exclusion of most of the individuals resulted in a knife-edge pattern of the escape behaviour for cod, which suggested that all length dependency in the escape behaviour occurred at one length. This, however, has little biological meaning. We could have used data or model smoothing, but we chose not to as this procedure is not recommended for the type of analysis we used [30].

In catch comparison studies, collecting bags or covers, which could quantify the difference between catches, generally are not used. As we were not able to quantify directly the escapement through the 115 mm standard trawl in this study, it is natural to wonder whether other mechanisms could produce the observed catch comparison pattern. In theory, extensive sex-related differences in length at age coupled with ontogenetic differential swimming ability could result in a cup-shaped escapement pattern. However, no such differences have been reported for cod and haddock. Moreover, accounting for the potential selectivity in the 115 mm meshes removed the cup-shaped pattern from the catch comparison curve for both cod and haddock and suggested a behavioral pattern that is in line with previous observations of the vertical preferences of fish inside the trawl body [1], [3], [5], [9], [10], [11], [13], [14], [31]. The influence that trawl body selectivity can have on the catch comparison curve was further illustrated by the simulation of the process with and without this selectivity in the trawl body.

Studies of behaviour in the trawl mouth have shown that haddock and saithe rise above the ground gear as they tire, whereas flatfish, cod, and *Nephrops* enter the trawl closer to the sea bed [9], [31], [33]. These observations of vertical preferences in the trawl mouth are similar to the patterns of escape behaviour we found in the main body of the trawl in the current study. The tendency for fish to exhibit varying degrees of rising in the trawl has led to the development of multi-level trawls equipped with horizontal separators and multiple codends, which allow partial segregation of the catch by species [5], [9]–[11], [14]–[15], [31]. The rather limited observations of cod in trawl nets indicate that they drift slowly back towards the codend, staying stationary in the net for long periods of time [34]–[35]. Thomsen [35] made underwater observations inside a trawl and reported that cod tend to rise as other gadoids such as haddock and whiting do; however, their rate of ascent was far slower and further aft in the trawl compared to that of other gadoids. Krag et al. [5], [14] conducted behavioural studies with trawl designs identical to those used in the current study. These behavioural studies focused on the section of the trawl where the aft tapered section is joined to the extension, which is equivalent to where the aft end of the large mesh panel was situated in the current study. Krag et al. [14] divided the extension into three vertically stacked compartments. In the upper half of the extension (upper compartment), 54% of the cod, 87% of the haddock, and 50% of the lemon sole were caught. The same separation device was used in another study by Krag et al. [5] that was designed to compare direct and indirect observations of fish behaviour. Similar catch proportions were found, with 57% of the cod, 73% of the haddock, and 39% of the lemon sole caught in the upper compartment. However, length-dependent catch values were not given in either study. The escape behaviour values found in the current study for the large mesh panel are very similar to the catch values reported by Krag et al. [5], [14] for the upper half of the extension. These results support the assumption that not all fish come in contact with the large mesh panel. The comparable catch and escape proportions between Krag et al. [5], [14] and the current study indicate that the fish that meet the panel escape through it.

For a fish to escape through a large mesh panel or similar selective devices, it needs to come in contact with the panel. The 800 mm mesh size used in this study indicated the selective potential of large mesh panels in the aft tapered section of the trawl, as fish of all sizes could escape through the meshes. In general, there was a large effect of the panel for gadoids, a smaller effect for flatfish, and little effect for *Nephrops*. Large mesh panels could therefore be used to reduce the relatively large by-catches that are a common problem in the *Nephrops* directed fisheries [36]–[37]. Earlier reports of *Nephrops* behaviour [32], [38], which state that *Nephrops* are associated with the gear’s lower part and often are observed rolling along the lower panel in the trawl, are in line with the results of this study. *Nephrops* use most of their energy in front of the trawl trying to out-swim the trawl using rapid tail flicks [39], which may explain the more passive behaviour of *Nephrops* once they are inside the net. The selectivity of *Nephrops* in the trawl body seems to be determined solely by the trawl’s lower panel, whereas the opposite may be true for most fish. In areas and fisheries where the catch of *Nephrops* makes up the majority of the catch value, large meshes could be used in the entire upper panel of the trawl body and wings. Such a design would improve the species selectivity in the fishery and reduce the drag of the gear, thereby saving fuel without a significant effect on the catch of *Nephrops.* However, as *Nephrops* of all sizes potentially can escape through the 115 mm mesh size in the panel of the standard trawl that corresponds to the large mesh panel in the experimental trawl, our approach, in which the selectivity in the 115 mm is excluded, cannot be used for *Nephrops.*

The large mesh panel in this study was 12 m long (stretched length) and covered the length of the entire aft tapered section of the trawl. This section was gradually reduced from a diameter of about 1.4 m to about 0.7 m. Thus, the cross-sectional area of the inner volume was reduced by approximately 75%. This substantial but gradual reduction should have given most sizes of fish an opportunity to escape through the large meshes. If the fish felt threatened in the aft tapered section and perceived the large meshes as an escape opportunity, we would have caught very few fish, as was observed for saithe and haddock. However, this was not the case for cod and witch flounder, for which the contact was significantly lower. This means that the large mesh panel is significantly less efficient in reducing the catch of cod compared to haddock and saithe. Underwater observations in the narrow, aft end of the tapered section of a trawl design similar to the one we use [40] have shown that fish, also large fish, maintain a safe distance to the netting wall so that they do not come in direct contact with the netting. It is possible that the more ordered herding process, whereby the fish orientate themselves relative to the netting and maintain a safe distance from it, might become less ordered in small volumes (e.g., at the end of the aft tapered trawl section) and be replaced by a panic reaction. Several parts of the behavioural process remain poorly understood; however, more detailed information about the process could be used to improve the efficiency of selective devices. Quantitative indirect behaviour studies such as this one in combination with direct observation techniques have the potential to generate this information.

## Supporting Information

Appendix S1 **In this appendix we derive the formulas used to estimate the contact likelihood** ***c_l_*** **with the large mesh panel for a fish of length** ***l*** **.**
(DOCX)
