# Correction: Inferring Fish Escape Behaviour in Trawls Based on Catch Comparison Data: Model Development and Evaluation Based on Data from Skagerrak, Denmark

**DOI:** 10.1371/journal.pone.0100605

**Published:** 2014-06-20

**Authors:** The PLOS ONE Staff

The images for Figures 3 and 4 are incorrectly switched. The image that appears as Figure 3 should be Figure 4, and the image that appears as Figure 4 should be Figure 3. The figure legends appear in the correct order. Please see the corrected Figure 3 here.

**Figure 3:**
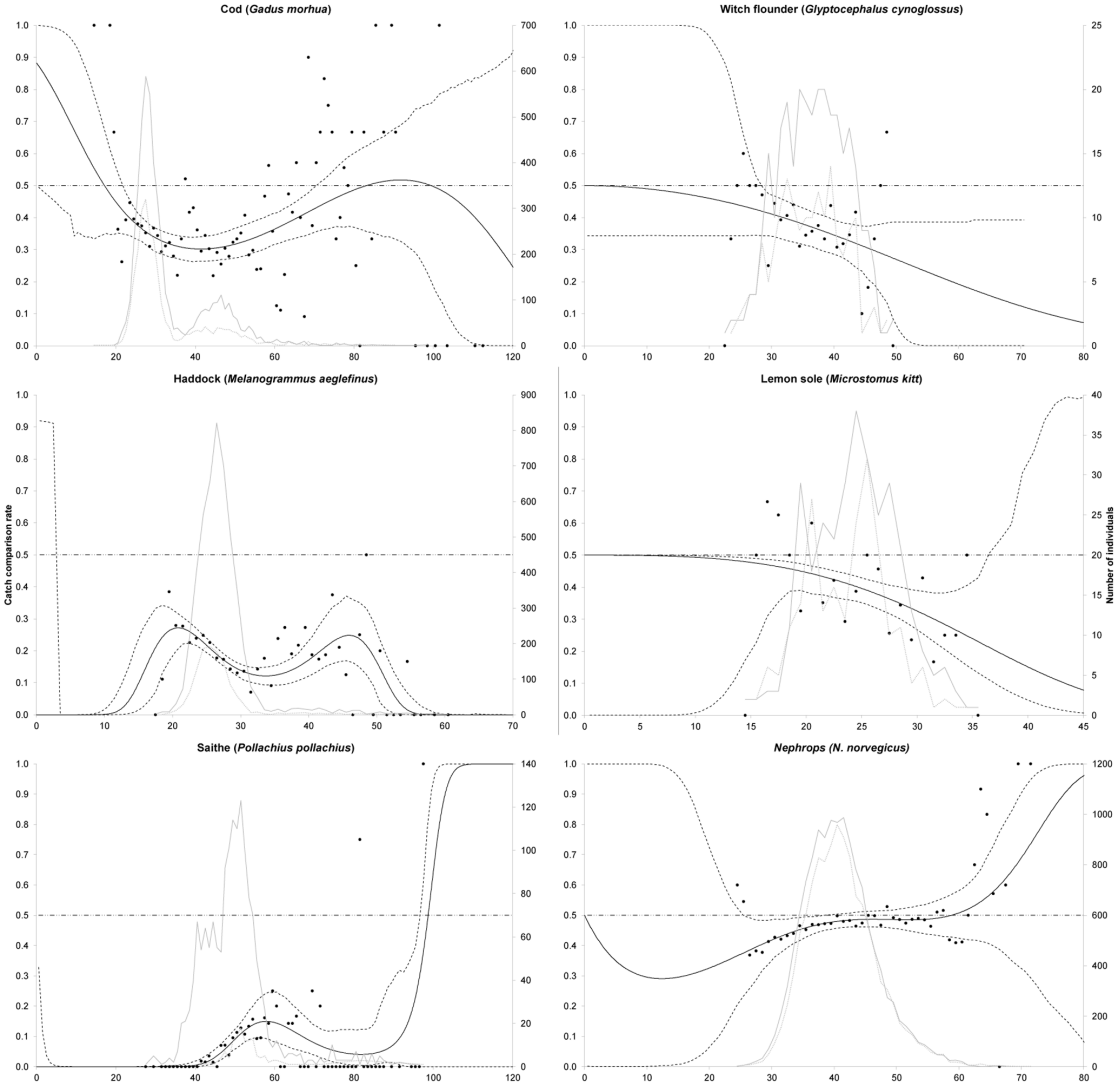
Catch comparison analysis and populations retained in both the experimental and standard trawls. Solid lines are mean estimates, and dotted lines indicate 95% confidence limits.

Please see the corrected Figure 4 here.

**Figure 4:**
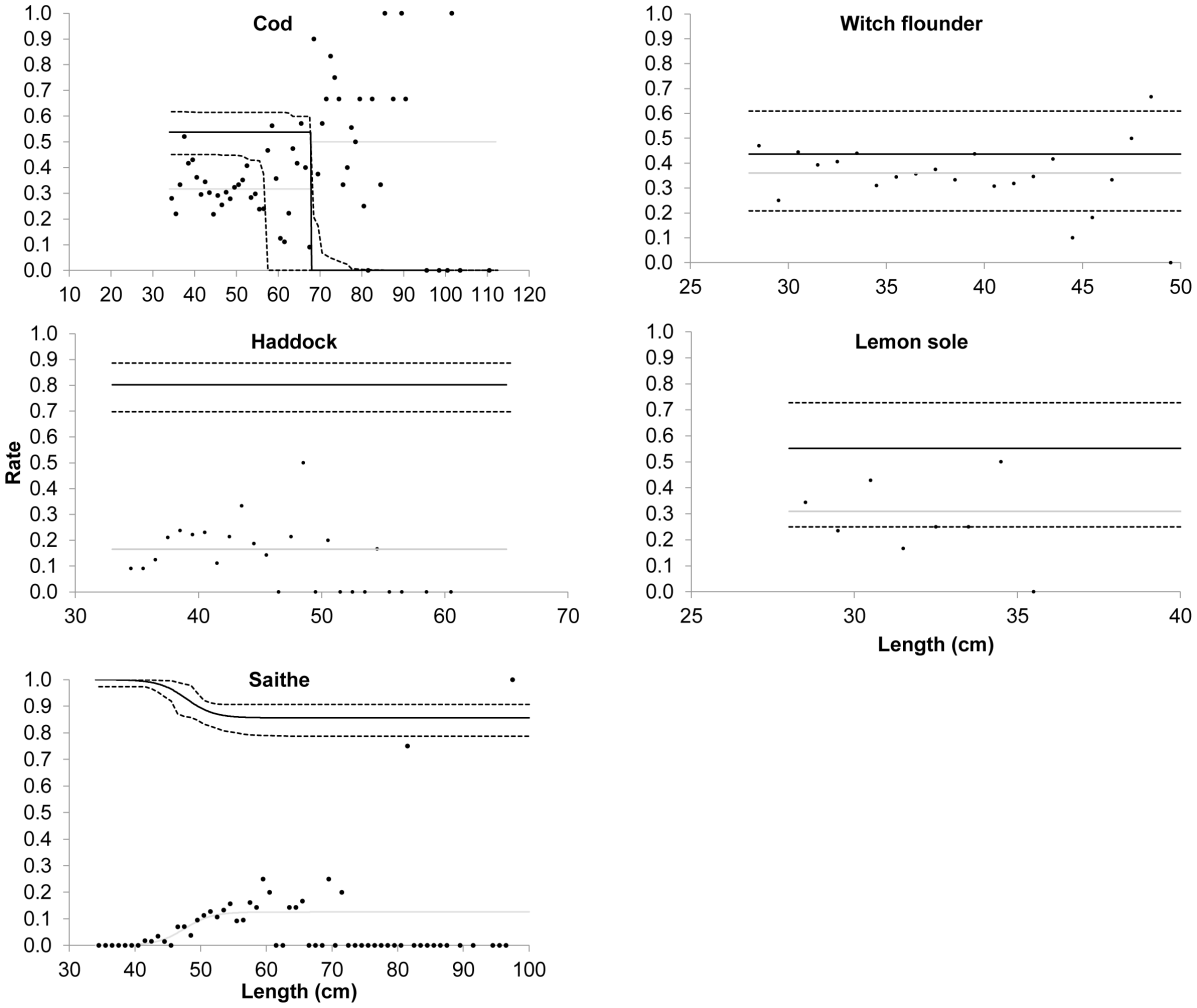
Estimated average escape behaviour (contact rate) (solid black curve) ±95% confidence limits (broken black curves), estimated mean retention (grey curve), and length-based retention data (black dots). Only length classes included in the catch and that could not escape through the 120 mm nominal mesh size were included in the modelling for all species.
