# Supplementary material for: Inferring Fish Escape Behaviour in Trawls Based on Catch Comparison Data: Model Development and Evaluation Based on Data from Skagerrak, Denmark
Source: PLoS One. 2014 Feb 20;9(2):e88819. doi: 10.1371/journal.pone.0088819 (PMC3930632; doi:10.1371/journal.pone.0088819)
Supplement: Appendix S1 — In this appendix we derive the formulas used to estimate the contact likelihood cl with the large mesh panel for a fish of length l . (DOCX) [file pone.0088819.s001.docx]

## Appendix

In this appendix we derive the formulas used to estimate the contact likelihood *c_l_* with the large mesh panel for a fish of length *l*. The estimation is based on the observed catch comparison rate *rate_l_*_._ Since the catch comparison rate is based on comparing the amount of fish of a certain size *l* found in the two codends (*nr1_l_* and *nr2_l_*), we first model how these values depend on the number of fish *n_l_* of a given length *l* in the population available to enter the two trawl bodies, and on the size selection processes in the different sections of the two trawls. The designs of the two legs in the twin trawl are identical except of section *j* (Figure A1):

section *j*

section *q*

section 1

*nr2_l_*

*n2_l_*

*n_l_*

*sp*

*1-sp*

*nr1_l_*

*n1_l_*

Figure A1. Drawing of the twin trawl design used in the experimental study.

Entry into the two legs is defined by the assumed length-independent split, $sp$:

$\begin{matrix} n1_{l}=sp\times n_{l} \\ n2_{l}=(1.0-sp)\times n_{l} \end{matrix}$ (A1)

For a fish entering one of the trawl bodies to end up being retained in codend of the specific trawl body requires that it does not escape during its travel down through the different sections of the trawl body before reaching the codend. Thus, the likelihood a fish of length *l* entering section *j* requires that it is retained by the preceding section/s. This can be modelled by the size selection *r1_1l_* in the first section of the experimental trawl, and by the size selection *r1_2l_* in the second section of the trawl. Thus, the likelihood for the fish entering the third section, given it entered the preceding sections, can be expressed by the product *r1_1l_* × *r1_2l_* of the size selection in the two preceding sections. Using this stepwise approach for a trawl body consisting of *q* sections (including codend) the likelihood for the fish to be found in the codend given it entered the preceding section/s can be expressed by the product *r1_1l_* × *r1_2l_* ×...× *r1_(q-1)l_* × *r1_ql_* of the size selection in each of the *q* sections. Using standard mathematical notation, this product is written as:

$r1_{1l}\times r1_{2l}\times...\times r1_{(q-1)l}\times r1_{ql}=\prod_{i=1}^{q} r1_{il}$ (A2)

Using the above approach and the mathematical notation given by (A2), the number of fish of size *l* found in each of the two codends can be expressed by:

$\begin{matrix} nr1_{l}=n1_{l}\times\prod_{i=1}^{q} r1{}_{il}=n1\times sp\times\prod_{i=1}^{q} r1_{il} \\ nr2_{l}=n2_{l}\times\prod_{i=1}^{q} r2_{il}=n_{l}\times(1-sp)\times\prod_{i=1}^{q} r2_{il} \end{matrix}$ (A3)

where ${r1}_{il}$ is the retention likelihood for a fish entering section $i$ in leg $1$ given that it enters the section. Thus, formula (A3) simply expresses that *nr1_l_* and *nr2_l_* can be estimated based on the number of fish entering each of the two legs of the trawl multiplied by the retention likelihood for each of the sections in the trawl leg in question. Except for section *j*, the designs of the two legs are identical. Therefore, we can assume:

$r1_{il}=r2_{il};\forall i\neq j$ (A4)

Therefore, (A3) can be written as:

$\begin{matrix} nr1_{l}=n_{l}\times sp\times r1_{jl}\times\left(\prod_{i=1}^{j-1} r1_{il}\right)\times\left(\prod_{i=j+1}^{q} r1_{il}\right) \\ nr2_{l}=n_{l}\times(1-sp)\times r2_{jl}\times\left(\prod_{i=1}^{j-1} r1_{il}\right)\times\left(\prod_{i=j+1}^{q} r1_{il}\right) \end{matrix}$ (A5)

Where we have used that the retention likelihood in the corresponding sections of the two trawl legs can be assumed to be identical except from in the section containing the large mesh panel.

Using (A5), the catch comparison ${rate}_{l}$ can be expressed by:

$\begin{matrix} rate_{l}=\frac{nr1_{l}}{nr1_{l}+nr2_{l}}= \\ \frac{n_{l}\times sp\times r1_{jl}\times\left(\prod_{i=1}^{j-1} r1_{il}\right)\times\left(\prod_{i=j+1}^{q} r1_{il}\right)}{n_{l}\times sp\times r1_{jl}\times\left(\prod_{i=1}^{j-1} r1_{il}\right)\times\left(\prod_{i=j+1}^{q} r1_{il}\right)+n_{l}\times(1-sp)\times r2_{jl}\times\left(\prod_{i=1}^{j-1} r1_{il}\right)\times\left(\prod_{i=j+1}^{q} r1_{il}\right)} \end{matrix}$ (A6)

Inspecting (A6), we see that each term in both the nominator and the denominator is multiplied by:

$n_{l}\times\left(\prod_{i=1}^{j-1} r1_{il}\right)\times\left(\prod_{i=j+1}^{q} r1_{il}\right)$ (A7)

Reducing (A6) by (A7) leads to:

$rate_{l}=\frac{sp\times r1_{jl}}{sp\times r1_{jl}+(1-sp)\times r2_{jl}}$ (A8)

Thus, the catch comparison rate can be reduced to modelling the size selection in section $j$ of the two legs in the twin trawl even though we have considered the potential size selection in each of the trawl sections of the two trawl legs. In leg 1, section $j$ consists of an upper and a lower panel with mesh size of 800 mm and 120 mm, respectively. While in leg 2, section *j*'s upper and lower panels both have a mesh size of 120 cm. If we restrict the analyses to a size range of fish above the point at which the 120 mm panel can be selective and below the point at which the large mesh panel can begin to restrict escapement, we have:

$\begin{matrix} r1_{jl}=1-c_{l} \\ r2_{jl}=1 \end{matrix}$ (A9)

where *c_l_* is the likelihood that a fish of length *l*, which enters the large mesh panel section, actually comes into contact with the panel and escapes. Inserting (A9) into (A8) leads to:

$rate_{l}=\frac{sp\times(1-c_{l})}{1-sp\times c_{l}}$ (A10)

(A10) is similar to formula (4).

To include fish of sizes *below* the point at which the 120 mm panels of trawl section *j* in both the legs are fully selective, we also need to consider the effect of the size selectivity in the 120 mm mesh panels on the catch comparison rate. Thus, the following formula can be applied:

$\begin{matrix} r1{}_{ij}=1-c_{l}-\log it\_M120_{l}\times(1-c_{l})\times c\_low_{l} \\ r2_{jl}=1-c_{l}\times\log it\_M120_{l}-\log it\_M120_{l}\times(1-c_{l}\times\log it\_M120_{l})\times c\_low_{l} \end{matrix}$ (A11)

where we assume that the size selectivity of each of the 120 mm panels can be modelled by the same *logit* function [29] (*logit_M120_l_*) for the fish actually contacting that panel. *c_low_l_* is the contact likelihood with the lower panels in section *j* given that the fish has not escaped through the upper panel in that section. Thus, for this situation, (A11) needs to be applied in (A8).
